# The FvHOG1 pathway is essential for stress responses, fungicide resistance, fumonisin B1 production and pathogenesis in *Fusarium verticillioides*

**DOI:** 10.1007/s44297-025-00052-5

**Published:** 2025-06-27

**Authors:** Haoxue Xia, Xulin Li, Yaru He, Wende Liu, Guangfei Tang

**Affiliations:** State Key Laboratory for Biology of Plant Diseases and Insect Pests, Institute of Plant Protection, Chinese Academy of Agricultural Sciences, Beijing, China

**Keywords:** Hog1-MAPK pathway, Fumonisin B1, Pathogenicity, *F. verticillioides*

## Abstract

The high-osmolarity glycerol (Hog) 1 signaling pathway is a component of mitogen-activated protein kinase (MAPK) cascades, which govern the responses of organisms to a wide variety of extracellular stimuli and assume a pivotal role in modulating growth, development, and pathogenicity across various organisms. However, the functions of Hog1-MAPK pathway proteins and the underlying mechanisms governing their development and pathogenicity remain largely unclear in *Fusarium verticillioides*. In this study, we investigated the role of all five Hog1-MAPK pathway genes (*FvSln1*, *FvSho1*, *FvSsk2*, *FvPbs2* and *FvHog1*) in *F. verticillioides*. Target gene deletion demonstrated that *FvSln1* and *FvSho1* play a minor role in the response to osmotic stress, but the virulence of Δ*FvSho1* in maize is impaired. The *FvSsk2*, *FvPbs2* and *FvHog1* mutants all presented a reduction in conidiation. These mutants are not only hypersensitive to osmotic stress but also show increased tolerance to oxidative stress, plasma membrane stress, and cell wall stress. The *FvSsk2*, *FvPbs2* and *FvHog1* mutants led to impairments in FB1 biosynthesis and pathogenicity. Interestingly, these mutants also have increased tolerance to fungicides. Overall, the findings of our study suggest that the FvSsk2-FvPbs2-FvHog1 MAPK cascade plays a crucial role in modulating conidiation, FB1 production, plant infection, and responses to hyperosmotic and general stresses in *F. verticillioides.*

**Supplementary Information:**

The online version contains supplementary material available at 10.1007/s44297-025-00052-5.

## Introduction

*Fusarium verticillioides* (*F. verticillioides*) is a widely distributed phytopathogen. It can incite a variety of devastating diseases that cause corn stalk rot and ear rot worldwide, thereby posing a severe threat to global corn yields and quality because of its production of multiple mycotoxins [1]. Among the mycotoxins produced by *F. verticillioides*, fumonisin B1 (FB1) stands out as the most frequently detected and comprehensively studied mycotoxin in maize crops affected by this pathogen [2]. The World Health Organization (WHO) previously reported that approximately 50% of the world's maize and maize-derived products are contaminated by FB1 to varying degrees [3]. FB1 has been classified as a potential carcinogen by the International Agency for Research on Cancer [4]. Research indicates that long-term consumption of foods contaminated with fumonisin may be associated with the occurrence of certain human cancers, such as esophageal cancer and liver cancer [5]. FB1 serves as a potent inhibitor of ceramide synthase, which is a pivotal enzyme in the biosynthesis of sphingolipids [6]. In *F. verticillioides*, the *FUM* gene cluster, which encompasses all the structural genes necessary for the biosynthesis of FB1, has been identified and comprehensively characterized [7]. Therefore, deciphering the molecular mechanisms of pathogenicity and FB1 synthesis in *F. verticillioides* is highly important for disease prevention and control.

Genetic investigations of* F*. *verticillioides* have pinpointed a diverse array of genes. These genes include *FUM* genes [8], G proteins [9], cAMP‒PKA genes [10], protein kinases [11], phosphatases [12] and a range of other genes. Collectively, these genes govern numerous biological processes within* F*. *verticillioides*. These processes include hyphal growth, the formation of conidia, the production of FB1, the response to stress, and the mechanisms underlying pathogenesis. The availability of the genome sequence of *F*. *verticillioides* has laid a solid foundation for the functional characterization of genes that are crucial for fungal biology [13]. This foundation enables researchers to utilize targeted mutagenesis techniques to explore and understand the roles of these genes more comprehensively. Over the past decade, numerous genes, such as *FvMK1*, *FvSTUA*, *FvMbp1*, *FvCpsA*, and *FvVE1*, have been demonstrated to affect the production of FB1 and pathogenicity in *F. verticillioides* [14–18]. An in-depth understanding of the pathogenic mechanism of* F*. *verticillioides* can offer approaches and potential targets for the prevention and control of *Fusarium* disease.

The capacity of fungal cells to rapidly adapt to a hostile environment hinges on a swift and robust stress response mechanism [19, 20]. The mitogen-activated protein kinase (MAPK) pathway lies at the core of the cellular signaling cascade. This cascade is responsible for transmitting diverse cellular signals and plays a crucial role in regulating fungal growth, development and pathogenicity [21]. MAPK pathways are composed of a module consisting of three kinases, namely, MAP kinase kinase kinase (MAPKKK), MAP kinase kinase (MAPKK), and MAP kinase (MAPK). In response to various signals, they are activated via sequential phosphorylation [22]. Among these MAPK cascades, the high osmolarity glycerol (Hog) 1 mitogen-activated protein kinase pathway plays a conserved role in osmoregulation across various organisms [23]. In the budding yeast *Saccharomyces cerevisiae*, the Hog response pathway, which is composed of the Ssk2/Ssk22-Pbs2-Hog1 cascade, is crucial for yeast survival under hyperosmotic conditions. Two transmembrane osmosensors, Sln1 and Sho1, have been recognized. These osmosensors can independently trigger the activation of the Hog1-MAPK pathway in response to an increase in external osmolarity [24]. In filamentous fungi, the key components of the Hog1-MAPK pathway are highly conserved. Nevertheless, their biological functions are not confined to responses to high osmolarity alone. In general, orthologs of Hog1 have been shown to be involved in regulating plant infection, conferring fungicide resistance, and mediating responses to diverse environmental stresses [25]. However, the specific functions of the Hog1-MAPK pathway vary considerably among different fungal pathogens.

The Hog1-MAPK signaling cascade has been reported in different filamentous fungi. For example, the *FgHog1* mutant displays impairments in both plant infection and the production of deoxynivalenol (DON) in *Fusarium graminearum* [26]. In *Ustilaginoidea virens*, UvHog1 is involved in regulating the biosynthesis of secondary metabolites that are toxic to plant cells [27]. However, in *Magnaporthe oryzae*, the *osm1* deletion mutant is deficient in the osmoregulation of hyphae. However, it functions normally in the generation of appressorium turgor and the process of plant infection [28]. Like in *M. oryzae*, the Hog1/Osm1 ortholog is not required for plant infection in several fungal pathogens, including *Cochliobolus orbiculare* and *Bipolaris oryzae* [29, 30]. The membrane-spanning proteins Sho1 and Sln1, which serve as biosensors of the Hog1-MAPK pathway, have also been functionally characterized. The Sln1 ortholog in *Candida albicans*, CaSln1, participates in the process of hyphal formation and contributes to the virulence of this fungus [31]. In *Botrytis cinerea*, BcSHO1 and BcSLN1 display certain functional redundancy in regulating fungal development, pathogenesis, and the response to osmotic stress [32]. Furthermore, in* F*. *graminearum*, FgSho1 is involved in cell wall-related processes rather than the response to osmotic stress [33]. In contrast, the *TcsB* mutant of *Aspergillus fumigatus* (which is an ortholog of Sln1) did not exhibit any observable phenotypic alterations [34]. Therefore, the Hog1-MAPK pathway likely plays species-specific roles during plant infection. Recently, our findings also demonstrated that FvHog1 is essential for the biosynthesis of fumonisin B1 (FB1), pathogenicity, and maintenance of Ca^2+^ homeostasis in *F. verticillioides* [2]. Although individual Hog1-MAPK pathway genes have been characterized for their regulatory roles in pathogenesis, development, and stress responses in a number of plant pathogenic fungi, to date, no detailed reports on Hog1-MAPK pathway gene mutants in *F. verticillioides exist*. Here, we comprehensively identified all the components of the Hog1-MAPK cascade in *F. verticillioides*. Through gene disruption experiments, we systematically characterized their functions in aspects such as conidiation, stress tolerance, and pathogenicity.

## Results

### Bioinformatic features of the key genes of the Hog1-MAPK pathway in *F. verticillioides*

The Hog1-MAPK pathway in *Saccharomyces cerevisiae* serves as a crucial mechanism for responding to changes in external osmolarity [35]. In our previous study, we characterized the function of Hog1. In this study, we aimed to characterize the Hog1-MAPK pathway in *F. verticillioides comprehensively*. Through a BLASTp search conducted on the NCBI database, using the corresponding protein sequences of *S. cerevisiae* as queries, orthologs of Sln1, Sho1, Ssk2, Pbs2, and Hog1 were successfully identified in *F. verticillioides*. The gene accession codes for *FvSln1*, *FvSho1*, *FvSsk2*, *FvPbs2*, and *FvHog1* in *F. verticillioides* are FVEG_02765, FVEG_03984, FVEG_00869, FVEG_01970, and FVEG_04168, respectively (Fig. S1a). The identified proteins were found to comprise 1150, 324, 1340, 654, and 306 amino acids, respectively. After these genes were identified, we further analyzed the domains of these proteins. FvSln1 contains a transmembrane domain at the N-terminus, a histidine kinase domain, an HATPase_c domain, and a response regulatory domain. FvSho1 contains four transmembrane domains and an SH3 domain. FvSsk2 and FvHog1 also contained an S_TKc domain. FvPbs2 contains a protein kinase domain (Fig. S1b). Sequence alignment and phylogenetic analysis revealed that the identified orthologous proteins are highly conserved among the fungi *F. verticillioides*, *F. graminearum*, *Magnaporthe oryzae*, *Zymoseptoria tritici*, *Botrytis cinerea,* and *S. cerevisiae* (Fig. S1c).

### The Hog1-MAPK pathway is required for conidiation in *F. verticillioides*

**Fig. 1:**
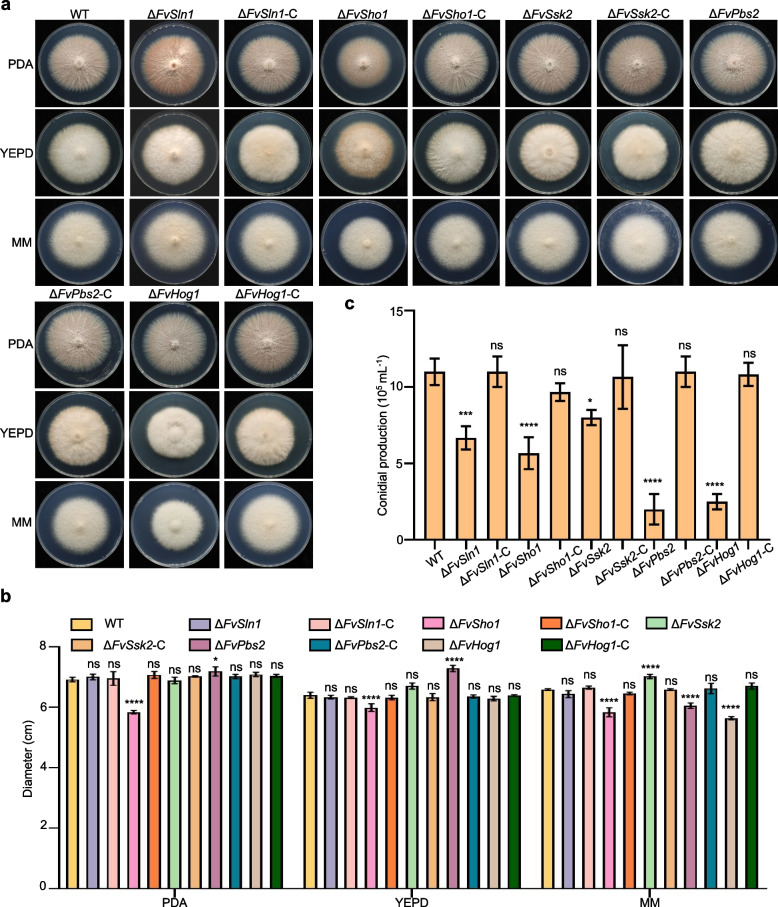
Effects of the deletion of key genes of the FvHog1-MAPK pathway on the growth and conidiation of *F. verticillioides*. **a** The WT, Δ*FvSln1*, Δ*FvSho1*, Δ*FvSsk2*, Δ*FvPbs2*, and Δ*FvHog1* strains and the complemented strains were grown on PDA, YEPD and MM media at 25 °C for 5 days. **b** Colony diameters of the WT, Δ*FvSln1*, Δ*FvSho1*, Δ*FvSsk2*, Δ*FvPbs2*, Δ*FvHog1* and complemented strains on PDA, YEPD and MM media were measured. **c** Conidiation of the WT, Δ*FvSln1*, Δ*FvSho1*, Δ*FvSsk2*, Δ*FvPbs2*, Δ*FvHog1* and complemented strains was measured. *P < 0.05, **P < 0.01, ***P < 0.001, ****P < 0.0001

To understand the roles of FvSln1, FvSho1, FvSsk2, FvPbs2, and FvHog1 in *F. verticillioides*, we generated gene deletion mutants (Fig. S2). To confirm that the defects observed in the deletion mutant strains were caused by gene deletion, a complementation strain was constructed by transforming a fragment containing the full-length gene and its native promoter into the deletion mutant strains via the pGTN vector. The complementation strain was verified by RT‒qPCR analysis (Fig. S2b). The growth patterns of the Δ*FvSln1*, Δ*FvSsk2*, Δ*FvPbs2*, and Δ*FvHog1* strains were similar to those of the wild type on PDA, YEPD, and MM media (Fig. 1a, b). The Δ*FvSho1* strain grew significantly more slowly than did the wild type (Fig. 1a, b). These findings suggest that FvSho1 plays a crucial role in vegetative growth.

To determine the effects of the deletion of genes related to the Hog1-MAPK pathway on conidiation, each strain was inoculated into PDA media and incubated at 25 °C for 5 days. As shown in Fig. 1c, all the deletion mutants presented severe defects in conidiation production compared with the wild type. These results suggest that the Hog1-MAPK pathway is not important for the hyphal growth of *F. verticillioides* but is closely associated with the production of conidia.

### The FvSsk2-FvPbs2-FvHog1 MAPK cascade is involved in the response to osmotic stress in *F. verticillioides*

**Fig. 2:**
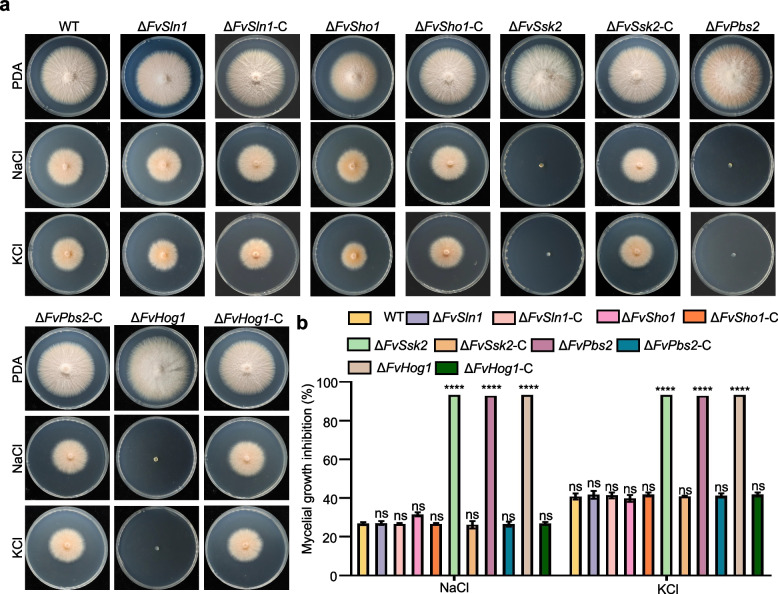
Effects of the deletions of *FvSln1*, *FvSho1*, *FvSsk2*, *FvPbs2 and FvHog1* on the response of *F. verticillioides* to osmotic stress. **a** Images of WT, deletion mutant and complemented strains grown at 25 °C for 5 days on PDA supplemented with NaCl and KCl. **b** Growth inhibition rates of the deletion mutant strains and complemented strains by NaCl and KCl were measured. *P < 0.05, **P < 0.01, ***P < 0.001, ****P < 0.0001

To explore the functions of the FvSln1, FvSho1, FvSsk2, FvPbs2, and FvHog1 in the response to osmotic stress in *F. verticillioides*, we compared the osmotic stress sensitivities of the Δ*FvSln1*, Δ*FvSho1*, Δ*FvSsk2*, Δ*FvPbs2*, and Δ*FvHog1* strains. The colony growth of the Δ*FvSsk2*, Δ*FvPbs2*, and Δ*FvHog1* strains was severely impeded by 0.7 M NaCl and 1 M KCl (Fig. 2). Intriguingly, we observed that, compared with the wild type, the Δ*FvSho1* and Δ*FvSln1* strains presented no significant alteration in their sensitivity to the osmotic stress induced by NaCl and KCl (Fig. 2). These findings suggest that FvSsk2, FvPbs2, and FvHog1 play crucial roles in the adaptation of *F. verticillioides to osmotic stress*, whereas FvSln1 and FvSho1 do not contribute significantly to this adaptive process.

### Roles of the key genes of the Hog1-MAPK pathway in multiple stress responses

**Fig. 3:**
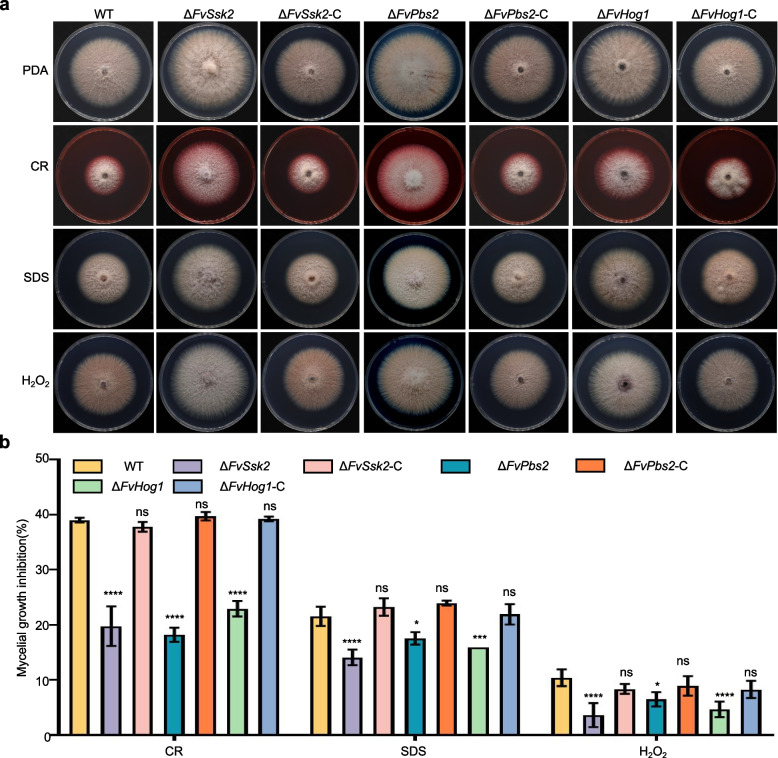
Key genes of the FvHog1-MAPK pathway are involved in the response of *F. verticillioides* to various stresses. **a** Mycelial growth of the WT, Δ*FvSsk2*, Δ*FvPbs2*, Δ*FvHog1*, and complemented strains on PDA supplemented with CR, SDS, and H_2_O_2_ was observed for 5 days. **b** Growth inhibition rates of the WT, Δ*FvSsk2*, Δ*FvPbs2*, Δ*FvHog1*, and complemented strains on PDA media under cell wall stress, plasma membrane stress and oxidative stress were measured. *P < 0.05, **P < 0.01, ***P < 0.001, ****P < 0.0001

To investigate whether the Hog1-MAPK pathway is involved in cell wall stress, plasma membrane stress and oxidative stress in *F. verticillioides*, we determined the sensitivity of the pathway mutants to Congo red (CR), SDS and H_2_O_2_. We found that the mycelial growth inhibition rates of the Δ*FvSho1* and Δ*FvSln1* strains under cell wall stress, plasma membrane stress and oxidative stress induced by CR, SDS, and H_2_O_2_, respectively, were essentially the same as those of the wild type. (Fig. S3a). Interestingly, we found that, unlike those of the Δ*FvSho1* and Δ*FvSln1 strains*, the growth of the Δ*FvSsk2*, Δ*FvPbs2*, and Δ*FvHog1* strains was less inhibited under CR, SDS, and H_2_O_2_ stress than that of the wild-type strain was, indicating that their tolerance to these stressors increased (Fig. 3a, b). These results indicate that FvSsk2, FvPbs2, and FvHog1 all play important roles in regulating the responses to cell wall stress, plasma membrane stress and oxidative stress.

### The FvSsk2-FvPbs2-FvHog1 MAPK cascade plays important roles in virulence and FB1 production

**Fig. 4:**
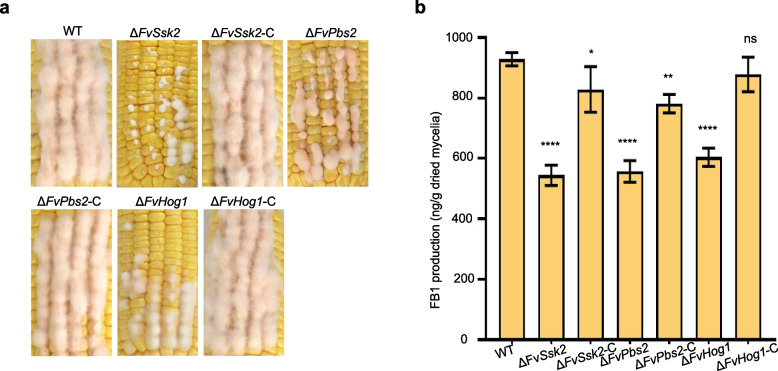
FvSsk2, FvPbs2, and FvHog1 are important for virulence and FB1 production. **a** Deletion mutants of *FvSsk2*, *FvPbs2*, and *FvHog1* presented significantly reduced virulence in the ears of corn. Infected corn ears were assessed after 7 days of inoculation with conidial suspensions of the WT, Δ*FvSsk2*, Δ*FvPbs2*, Δ*FvHog1*, and complemented strains. **b** Deletion mutants of *FvSsk2*, *FvPbs2*, and *FvHog1* presented significantly reduced FB1 production. The amount of FB1 produced by the WT, Δ*FvSsk2*, Δ*FvPbs2*, Δ*FvHog1*, and complemented strains was determined after growth in GYAM medium for 7 days. *P < 0.05, **P < 0.01, ***P < 0.001, ****P < 0.0001

The virulence of the Δ*FvSln1*, Δ*FvSho1*, Δ*FvSsk2*, Δ*FvPbs2*, and Δ*FvHog1* strains was assessed through the inoculation of conidial suspensions onto corn ears. At 7 dpi, the symptoms induced by the Δ*FvSho1*, Δ*FvSsk2*, Δ*FvPbs2*, and Δ*FvHog1* strains scarcely spread out from the inoculation site (Fig. 4a, Fig. S4a). In contrast, under identical conditions, both the wild-type and complemented strains resulted in the corn ears being densely covered with mycelium (Fig. 4a, Fig. S4a). The loss of *FvSln1* does not affect virulence (Fig. S4a). Given that FB1 has been identified as a virulence determinant of *F. verticillioides* during the corn seeding stage, we subsequently measured the production of FB1 in the deletion mutants. As shown in Fig. 4b and Fig. S4b, the production of FB1 by the Δ*FvSho1*, Δ*FvSsk2*, Δ*FvPbs2*, and Δ*FvHog1* strains significantly decreased, and the complementation strain successfully restored FB1 production to the level of the wild type. Notably, the deletion of *FvSln1* had no effect on the production of FB1 (Fig. S4b). Together, these findings strongly suggest that FvSho1, FvSsk2, FvPbs2, and FvHog1 play important roles in both virulence and FB1 production in *F. verticillioides*.

### The sensitivity of the key genes of the Hog1-MAPK pathway to fungicide stress

**Fig. 5:**
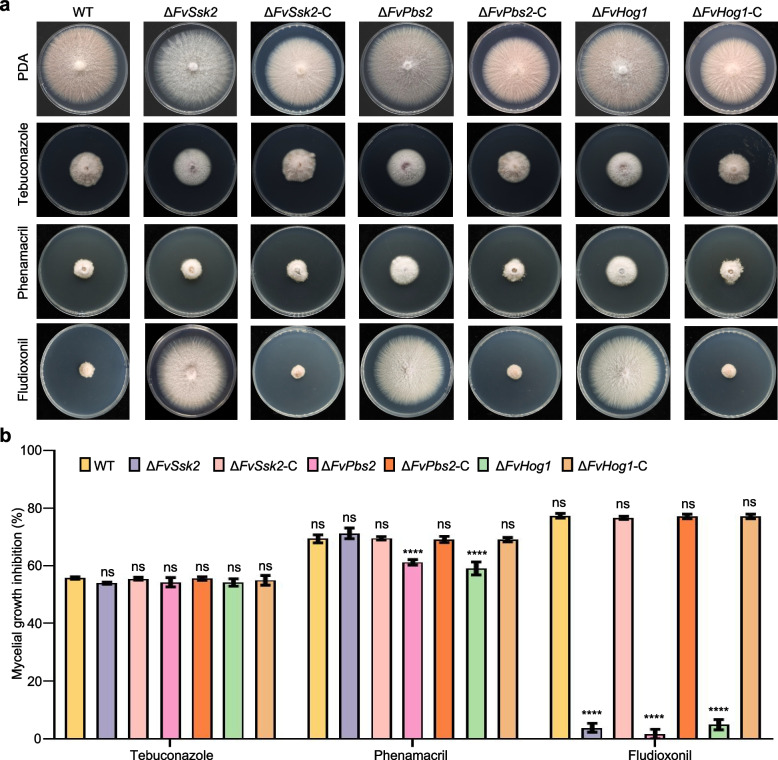
Sensitivity of the key genes of the Hog1-MAPK pathway in *F. verticillioides* to fungicides. **a** Mycelial growth of the WT, Δ*FvSsk2*, Δ*FvPbs2*, Δ*FvHog1* and complemented strains on PDA supplemented with fludioxonil, phenamacril, and tebuconazole at 25 °C for 5 days. **b** Growth inhibition rates of the WT, Δ*FvSsk2*, Δ*FvPbs2*, Δ*FvHog1* and complemented strains on PDA media under fungicide stress. *P < 0.05, **P < 0.01, ***P < 0.001, ****P < 0.0001

In agricultural production practices, fludioxonil, phenamacril, and tebuconazole are commonly used fungicides in the field. However, their extensive application has led to the development of potential resistance in pathogenic fungi [36, 37]. In terms of its biochemical mechanism of action, fludioxonil is thought to interfere with the Hog1-MAPK signaling pathway in fungi [38]. To investigate the sensitivity of key genes in the Hog1-MAPK signaling pathway to fungicides, we inoculated the mutant strains onto PDA plates containing fludioxonil, phenamacril, and tebuconazole. As shown in Fig. 5, the deletion of *FvSsk2*, *FvPbs2*, and *FvHog1* significantly increased resistance to fludioxonil. Compared with the wild-type strain, the Δ*FvPbs2* and Δ*FvHog1* strains also presented greater resistance to phenamacril (Fig. 5). In contrast, the loss of *FvSln1* and *FvSho1* had no obvious effect on the response to fludioxonil or phenamacril stress (Fig. S3b). The sensitivity of all the mutant strains to tebuconazole was not significantly altered. These results indicate that the Δ*FvSsk2*, Δ*FvPbs2* and Δ*FvHog1* strains are highly resistant to fludioxonil. FvPbs2 and FvHog1 are involved in the regulation of resistance to phenamacril.

## Discussion

The Hog1-MAPK pathway has species-specific functions in various physiological processes within phytopathogenic fungi [39]. *F. verticillioides* represents a dominant fungal species that incites corn stalk rot and ear rot in grain crops globally [40]. The biological function of Hog1-MAPK in *F. verticillioides* is of great interest and merits in-depth investigation. The Hog1-MAPK pathway has been demonstrated to be essential for the development and growth of fungi in certain other fungal species. In *Cryphonectria parasitica*, the Δ*cpmk1* mutants presented decreased pigmentation and a reduction in conidiation [41]. In *B*. *cinerea*, BcSak1 was demonstrated to be involved in the formation of conidia and appressoria-like structures [42]. In a previous investigation of *Bipolaris sorokiniana* Δ*Cshog1* mutants, no such phenotypic changes were discerned [43]. Nevertheless, in the present study, we identified all the genes in the Hog1-MAPK pathway from *F. verticillioides.* The MAPK cascade orthologous to the yeast Hog1-MAPK pathway is also well conserved in *F. verticillioides*. As expected, FvSln1 (FVEG_02765), FvSho1 (FVEG_03984), FvSsk2 (FVEG_00869), FvPbs2 (FVEG_01970), and FvHog1 (FVEG_04168) both contained conserved domains (Fig S1b). Compared with those of the wild-type strain, the growth rates of most mutant strains in this pathway did not significantly differ (Fig. 1a, b). However, the production of conidia in these mutant strains significantly changed (Fig. 1c). Our findings revealed that, in comparison with orthologs previously characterized in other fungal species, the Hog1-MAPK pathway performs both analogous and distinct functions in regulating the growth and development of fungi.

Previous work has shown that the Hog1-MAPK pathway manifests its most phylogenetically conserved function in yeast and filamentous fungi by orchestrating responses to hyperosmotic stress [25]. Here, analysis of Hog1-MAPK pathway mutant strains revealed that the Δ*FvSsk2*, Δ*FvPbs2*, and Δ*FvHog1* strains presented strikingly acute sensitivity to high osmotic stress conditions in *F. verticillioides*, which is in line with the present observations (Fig. 2). However, the deletion of *FvSln1* or *FvSho1* did not affect hyperosmotic stress sensitivity. This finding implies that within *F. verticillioides*, the functions of FvSln1 and FvSho1 operate independently of the Hog1-MAPK pathway, a phenomenon that diverges considerably from the well-established understanding of budding yeast [44]. The deletion of *FgSln1* and *FgSho1* in *F. graminearum* also has no effect on sensitivity to hyperosmotic stress [33], which indicates that an additional signaling pathway might be initiated and/or potentiated to activate the Hog1-MAPK pathway in Fusarium. Interestingly, pathogenicity investigations revealed that the Δ*FvSho1* mutant presented significantly diminished virulence when tested in maize. In a similar vein, within *Ustilago maydis* and *M*. *oryzae*, *Sho1*-deficient mutants presented attenuated virulence capabilities [45, 46]. These findings suggest that orthologs of Sho1 play crucial roles in the pathogenesis of pathogenic fungi.

In filamentous fungi, evidence has demonstrated that the Hog1-MAPK pathway is also involved in regulating responses to various environmental stresses [25]. In *A. fumigatus* and *B. cinerea*, the vegetative growth of mutants with impairments in this conserved Hog1-MAPK, including the Δ*sakA* and Δ*bcsak1* mutants, was highly sensitive to H_2_O_2_ treatment [34, 42]. Compared with wild-type *F*. *graminearum*, the *Fghog1* mutant presented marginally increased sensitivity to H_2_O_2_ and SDS [26]. Compared with the wild type, the *Uvhog1* mutant of *U. virens* presented no substantial changes in sensitivity to H_2_O_2_ [27]. Previous studies revealed that *hog1* mutants present increased resistance to particular cell wall inhibitory substances, such as Congo red and calcofluor white, in *C. albicans*. This finding suggests a link between the hog1 gene and the processes of the cell wall integrity (CWI) pathway [47]. In this study, the Δ*FvSsk2*, Δ*FvPbs2* and Δ*FvHog1* mutants presented increased tolerance to H_2_O_2_, SDS (a cell membrane-damaging agent) and Congo red (a cell wall-damaging agent) (Fig. 3). Therefore, it is highly probable that the Hog1-MAPK pathway in *F. verticillioides* is also involved in regulating responses to diverse environmental stresses. This situation resembles what has been observed in several other filamentous fungi [48]. The regulation of cell wall stress and oxidative stress by Hog1-MAPK is likely related to the crosstalk between the MAPK pathways [49]. In addition, the pathogenicity of these mutants was significantly reduced in maize (Fig. 4a). Therefore, the loss of pathogenicity of these mutants in maize was likely attributable, at least in part, to alterations in response to various environmental stresses. This is because the growth defects of these mutants are not particularly severe. The specific mechanisms still require in-depth research.

Furthermore, the Δ*FvSsk2*, Δ*FvPbs2* and Δ*FvHog1* mutants presented resistance to the fungicide fludioxonil, and the Δ*FvPbs2* and Δ*FvHog1* mutants presented resistance to phenamacril. (Fig. 5). Consistent with our results, numerous studies have revealed the association between this pathway and fungicide resistance. In *F. graminearum*, the Hog1-MAPK signaling pathway confers resistance to phenylpyrrole and dicarboximide fungicides [50, 51]. A mutation in *SRM1*, which encodes a MAPK related to the yeast Hog1, resulted in moderate resistance to the dicarboximide fungicides iprodione and procymidone and to the phenylpyrrole fungicide fludioxonil with respect to mycelial growth. Iprodione and procymidone, along with the phenylpyrrole fungicide, affect mycelial growth [29]. Moderate resistance to fungicides has been demonstrated in *Neurospora crassa* (Δ*os2*) and *Colletotrichum lagenarium* (Δ*osc1*) [52, 53]. As alluded to earlier, Hog1-MAPK is typically indispensable for pathogenicity across a wide range of fungal pathogens. The finding that the activity of fungicides is achieved through the activation of Hog1-related MAPK pathways implies that other signaling cascades, including those associated with Hog1-related MAPKs, could be valuable targets for the development of novel fungicides.

## Materials and methods

### Fungal strains and culture conditions

In this study, strains of *F. verticillioides* were cultured on potato dextrose agar (PDA) media at 25 °C for 5 days. The FB1-inducing medium was the GYAM medium (0.24 M glucose, 0.05% yeast extract, 8 mM L-asparagine, 5 mM malic acid, 1.7 mM NaCl, 4.4 mM K_2_HPO_4_, 2 mM MgSO_4_, and 8.8 mM CaCl_2_; pH = 3.0). The colony morphology was assessed on PDA media, yeast extract peptone dextrose (YEPD) agar media, and minimal medium (MM) agar media.

### Phylogenetic and domain analyses

The amino acid sequences of Sln1, Sho1, Ssk2, Pbs2, and Hog1 were retrieved from the FungiDB (https://fungidb.org/fungidb/app). The domains of these proteins were subsequently predicted via SMART (http://smart.embl-heidelberg.de) [54]. To construct a phylogenetic tree, we first obtained the homologous amino acid sequences of the Sln1, Sho1, Ssk2, Pbs2 and Hog1 proteins in multiple species from the FungiDB and then aligned them via ClustalW. Using MEGA 7 software and the neighbor‒joining method, we successfully constructed a phylogenetic tree.

### Construction of gene deletion and complementation strains

The gene knockout strains of Δ*FvSln1*, Δ*FvSho1*, Δ*FvSsk2*, Δ*FvPbs2*, and Δ*FvHog1* were successfully constructed via the double-joint PCR technique and protoplast transformation technique. First, primers were used to amplify approximately 1500 bp regions upstream and downstream of the target gene, as well as the hygromycin (HYG) fragment (Table S1). The three fragments were subsequently fused together. The fused fragment was subsequently introduced into the protoplasts of the wild-type strain via the PEG-mediated protoplast transformation technique. After that, the gene knockout mutants were identified through PCR detection methods (Table S1).

To construct the complementary strains, the primers listed in Table S1 were used to amplify the open reading frame (ORF) region of the target gene and its promoter region via PCR. The amplified fragments were subsequently cloned and inserted into the pGTN vector containing G418 resistance via the ClonExpress II One Step Cloning Kit. The constructed complementary vector was subsequently transformed into the protoplasts of the gene knockout mutants, resulting in complementary strains. Finally, the transformants were screened with G418 to determine the strains that had been successfully transformed [55].

### Conidiation assays

Five mycelial plugs were excised from the edge of each tested strain that had been grown on PDA for 5 days. These plugs were then transferred into 5 mL of ddH₂O. The mixture was subsequently filtered through a three-layer cloth and centrifuged at 5,000 rpm for 5 min. The conidia were subsequently resuspended in 5 mL of ddH_2_O to obtain a conidial suspension. The number of conidia was quantified via a hemocytometer. Each strain had three biological replicates.

### Stress assay

All the strains were cultivated on PDA media supplemented with 0.7 M NaCl, 1 M KCl, 1.5 M sorbitol, 10 mM H_2_O_2_, 0.01% sodium dodecyl sulfate (SDS), and 10 g/L Congo red (CR). The cultures were then incubated at 25 °C for 5 days. Each strain had three biological replicates.

### Fungicide susceptibility assay

For the fungicide sensitivity assay, all strains were cultured on PDA media supplemented with 0.01 μg/mL fludioxonil, 0.05 μg/mL tebuconazole, or 3 μg/mL phenamacril. After inoculation, the strains were incubated at 25 °C for 5 days [56]. Each strain had three biological replicates.

### Fumonisin B1 production assay

To quantify the production of fumonisin B1 (FB1), all the tested strains were cultured in GYAM liquid media. These strains were incubated at 25 °C in a shaker at 150 rpm. After 7 days, the supernatant was obtained by filtering the mycelia through three layers of gauze. The concentration of FB1 in the supernatant was subsequently measured precisely via an ELISA kit (Meimian MM-9513701).

### Pathogenicity assay

In this study, to examine the virulence of each strain, we inoculated corn ears with fungal spore suspensions of each strain at a concentration of 10^6^ conidia/mL. First, the surfaces of the corn ears were carefully disinfected with 75% ethanol. Each corn kernel was subsequently individually wounded via a sterilized toothpick. The fungal spore suspension was subsequently inoculated onto the corn kernels. After the inoculated corn ears were cultured in an incubator at 25 °C for 7 days, we took photos of the corn ears and analyzed their disease symptoms. Each strain in this experiment had three biological replicates.

### Statistical analysis

All the experiments were conducted independently three times. The values are the means ± SDs of three biological replicates. Significance was determined by two-way ANOVA. *P < 0.05, **P < 0.01, ***P < 0.001, ****P < 0.0001.

## Supplementary Information

Supplementary Material 1.

Supplementary Material 2.

## Data Availability

The datasets and materials supporting the results are included within the article, and the work described has not been published before.
