# Supplementary material for: The FvHOG1 pathway is essential for stress responses, fungicide resistance, fumonisin B1 production and pathogenesis in Fusarium verticillioides
Source: Crop Health. 2025 Jun 27;3(1):14. doi: 10.1007/s44297-025-00052-5 (PMC12825977; doi:10.1007/s44297-025-00052-5)
Supplement: Supplementary file 1 — Supplementary Material 1. [file 44297_2025_52_MOESM1_ESM.pdf]

Supplementary information

The FvHOG1 pathway is essential for stress responses, fungicide resistance, fumonisin B1 production and pathogenesis in *Fusarium verticillioides*

Haoxue Xia[1], Xulin Li[1], Yaru He[1], Wende Liu[1*], Guangfei Tang[1*]

[1]State Key Laboratory for Biology of Plant Diseases and Insect Pests, Institute of Plant Protection, Chinese Academy of Agricultural Sciences, Beijin, China

*Correspondence: Guangfei Tang (tangguangfei@caas.cn) and Wende Liu (liuwende@caas.cn)

a

Protein	Gene ID
FvSln1	FVEG_02765
FvSho1	FVEG_03984
FvSsk2	FVEG_00869
FvPbs2	FVEG_01970
FvHog1	FVEG_04168

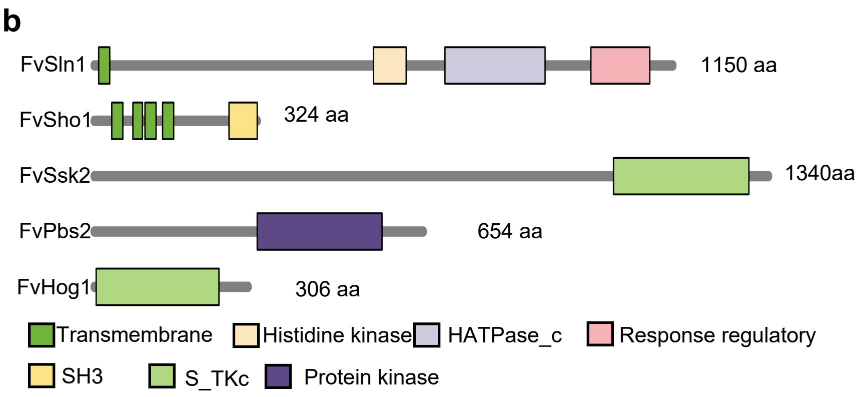

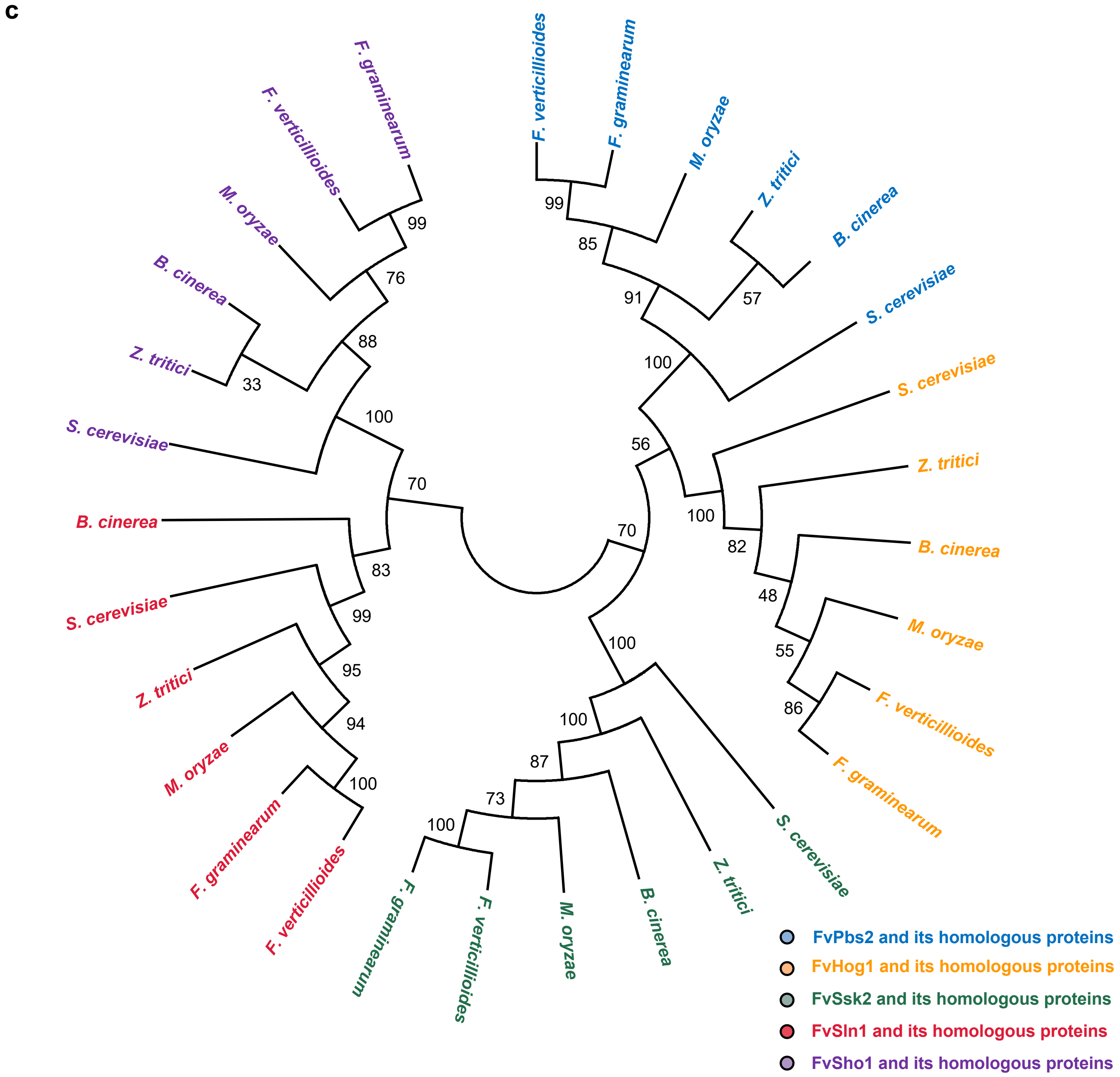

Fig. S1 Identification of the key genes of FvHog1-MAPK pathway in *F. verticillioides*. **(a)** The gene accession codes for FvSln1, FvSho1, FvSsk2, FvPbs2, and FvHog1 in *F. verticillioides*. **(b)** Conserved domain of FvSln1, FvSho1, FvSsk2, FvPbs2, and FvHog1 proteins. **(c)** Phylogenetic relationships of FvSln1, FvSho1, FvSsk2, FvPbs2, and FvHog1 proteins from various other fungi.

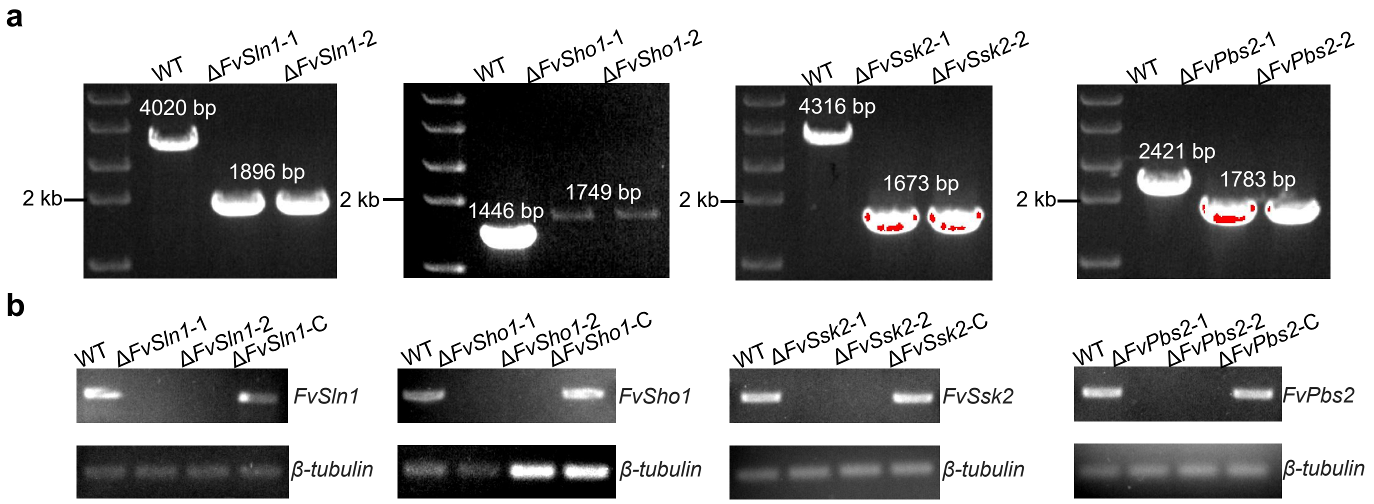

Fig. S2 Identification of Δ*FvSln1*, Δ*FvSho1*, Δ*FvSsk2*, and Δ*FvPbs2* strains in *F. verticillioides*. **(a)** PCR identification of Δ*FvSln1*, Δ*FvSho1*, Δ*FvSsk2* and Δ*FvPbs2*. **(b)** The expression level of *FvSln1*, *FvSho1*, *FvSsk2*, and *FvPbs2* gene was detected by RT-qPCR assays in WT, deletion mutants and complemented strains. *β-tubulin* served as the control.

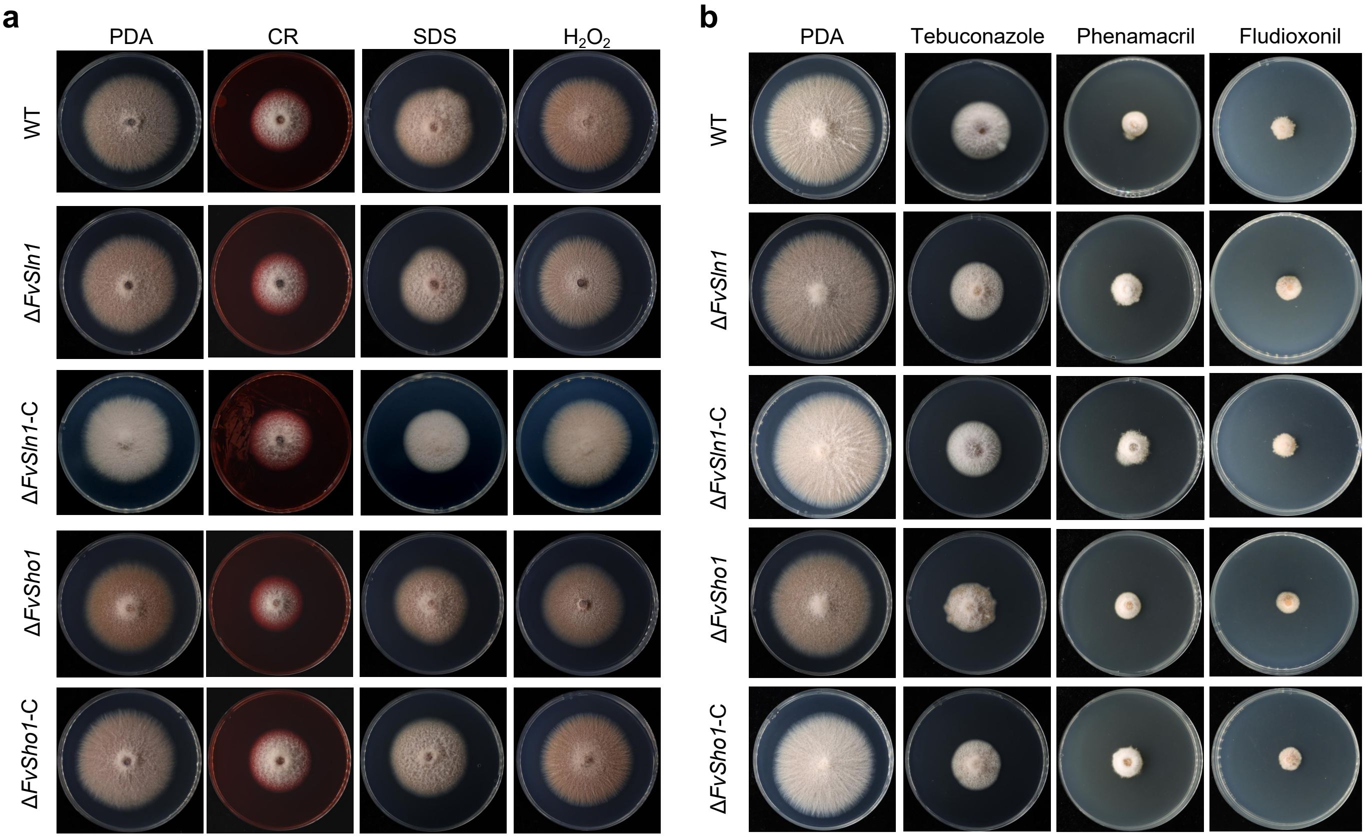

Fig. S3 The Δ*FvSln1* and Δ*FvSho1* are not involved in the response of *F. verticillioides* to various stresses. **(a)** Mycelial growth of the WT, Δ*FvSln1*, Δ*FvSho1* and complemented strains on PDA media under cell wall stress and oxidative stress were measured. **(b)** Mycelial growth of the WT, Δ*FvSln1*, Δ*FvSho1* and complemented strains on PDA media supplemented with fludioxonil, phenamacril, and tebuconazole at 25°C for 5 days.

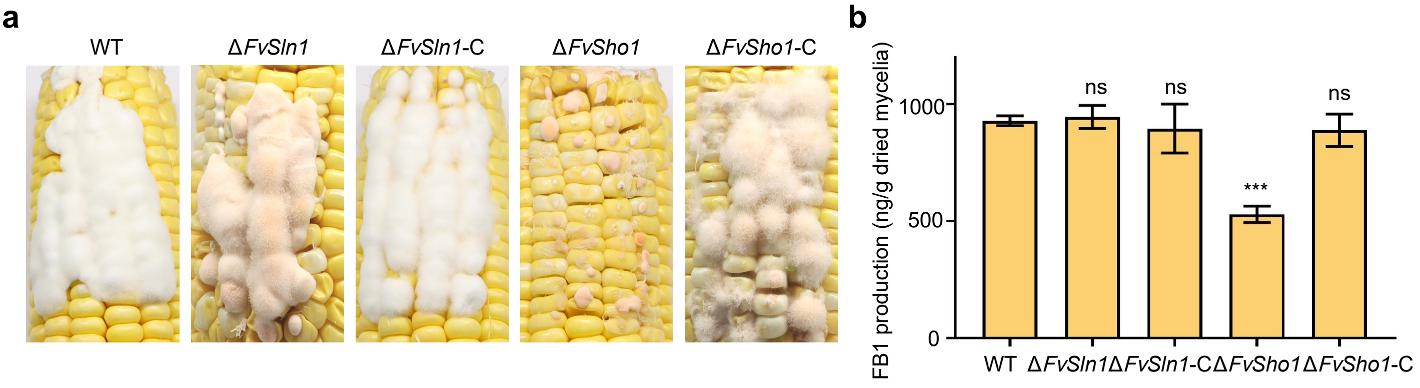

Fig. S4 FvSho1 is important for virulence and FB1 production. **(a)** Deletion mutants of *FvSho1* showed significantly reduced virulence on corn ears. Infected corn ears were assessed after 7 days of inoculation with conidial suspension of the WT, Δ*FvSln1*, Δ*FvSho1* and complemented strains. **(b)** Deletion mutants of *FvSho1* showed significantly reduced FB1 production. The amount of FB1 produced by the WT, Δ*FvSln1*, Δ*FvSho1*, and complemented strains was determined after growth in the GYAM medium for 7 days. *P < 0.05, **P < 0.01, ***P < 0.001, ****P < 0.0001.

Table S1 The primers listed in this study.
